# 1097. A Comparison of Area-Under Curve (AUC)-Guided vs Trough-Guided Monitoring of Vancomycin and Its Impact on Nephrotoxicity: A Systematic Review and Meta-analysis

**DOI:** 10.1093/ofid/ofab466.1291

**Published:** 2021-12-04

**Authors:** Ashley Shiyuan Lim, Jun Jie Benjamin Seng, Tao Tao Magdeline Ng, Hui Ting Chng, Zhe Han

**Affiliations:** 1 KK Women’s & Children’s Hospital, Singapore, Singapore; 2 Ministry of Health Holdings, Singapore, Singapore, Not Applicable, Singapore; 3 National University of Singapore, Singapore, Not Applicable, Singapore

## Abstract

**Background:**

Trough levels have been used for Vancomycin (VAN) therapeutic drug monitoring (TDM) historically due to its practicality. A paradigm shift towards the use of area under curve (AUC)-guided dosing TDM has been made due to availability of advanced pharmacokinetics software, variability between trough levels and AUC values and the potential for reducing toxicity. This review aims to evaluate the impact of AUC-guided vs trough-guided vancomycin TDM on nephrotoxicity-related outcomes.

**Methods:**

A systematic review was conducted using PubMed®, Embase®, Web of Science®, CINAHL®, Google scholar and Cochrane library® up till 1st January 2021 and was reported according to the PRISMA checklist. Studies which evaluated AUC-guided or trough-guided VAN TDM and vancomycin-associated nephrotoxicity were included. Random effects models were used to compare differences in nephrotoxicity between trough level or AUC based vancomycin TDM due to expected heterogeneity in study designs.

PRISMA Flowchart

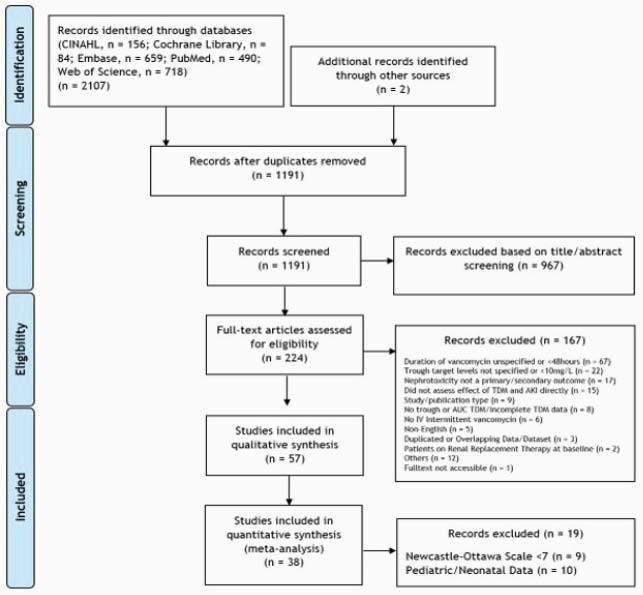

PRISMA flow chart depicting the selection process of studies included in the meta-analysis

**Results:**

Of 1191 records retrieved, 57 studies were included. Majority of studies included adult and elderly patients (n=47, 82.5%). The pooled prevalence of nephrotoxicity was lower using the AUC-guided TDM [6.2%, 95% confidence interval (CI): 2.9 – 9.5%] compared to trough-guided TDM [17.0%; 95% CI: 14.7 – 19.2%]. The risk of nephrotoxicity was lower with the AUC-guided approach as compared with the trough-guided approach [OR: 0.53, 95% CI: 0.32–0.89]. AUC thresholds correlated with risk of nephrotoxicity only for the first 96 hours of therapy. A frequency analysis of significant multivariable factors showed that concomitant use of nephrotoxins, VAN trough levels and duration of VAN therapy were most commonly associated with nephrotoxicity.

Forest plot comparing the risk of nephrotoxicity of AUC-guided vs trough-guided

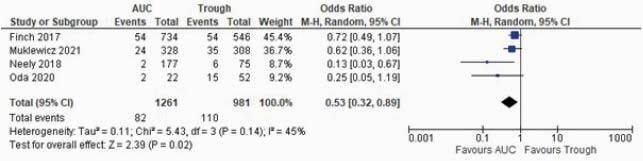

Forest plot comparing the risk of nephrotoxicity of AUC-guided vs trough-guided

Pooled nephrotoxicity rates from AUC-guided monitoring

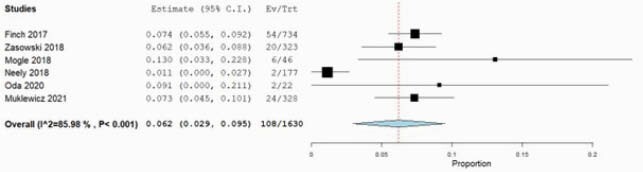

Pooled nephrotoxicity rates from AUC-guided monitoring

Pooled nephrotoxicity rates from trough-guided monitoring

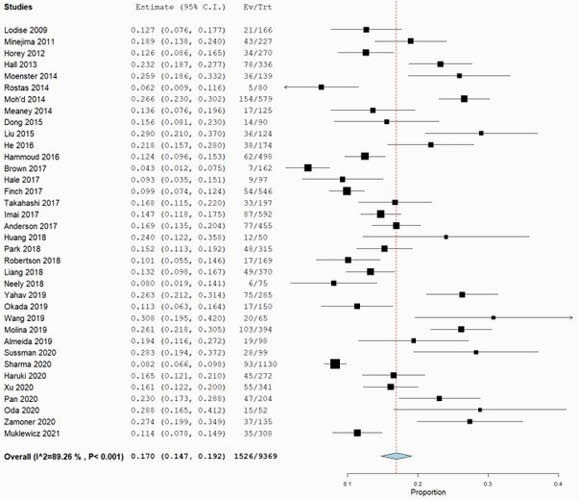

Pooled nephrotoxicity rates from trough-guided monitoring

**Conclusion:**

The AUC-guided approach appeared to have lower risk of nephrotoxicity which supports the updated American Society of Health-System Pharmacists recommendations. More studies should be performed to evaluate the optimal derivation of AUC and clinical utility of repeated measurements of vancomycin AUC and trough levels.

**Disclosures:**

**All Authors**: No reported disclosures

